# Hybrid-manufactured silicon nitride coated CFR-PEKK: A candidate biomaterial for trauma plate applications?

**DOI:** 10.1016/j.jmbbm.2025.107141

**Published:** 2025-07-17

**Authors:** Arjun Sharma, James A. Smith, Michael A. Kurtz, Tabitha Derr, Paul M. DeSantis, Ryan M. Bock, Steven M. Kurtz

**Affiliations:** a Implant Research Core, Drexel University School of Biomedical Engineering, Science, and Health Systems, Philadelphia, PA, USA; b SINTX Technologies, Inc., Salt Lake City, UT, USA

**Keywords:** Carbon-fiber-reinforced-polyetherketoneketone, CCF-PEKK, Trauma plates, Fused filament fabrication, Flexural testing

## Abstract

Continuous carbon fiber–reinforced polyetherketoneketone (CCF-PEKK) is a thermoplastic composite with properties suitable for trauma plate applications (elastic modulus, strength, radiolucency, and inertness). However, components manufactured by fused filament fabrication (FFF) often display non-uniform (anisotropic) mechanical properties and contain microstructural voids. To address these limitations, we investigated a hybrid-manufacturing approach, combining FFF with continuous carbon fiber reinforcement followed by uniaxial compression molding. Here, we asked: 1. Can layup orientation be tuned to replicate the mechanical stiffness of cortical bone in flexion? 2.) How does the structure of the different layups influence fracture behavior? and 3.) Do silicon nitride (Si_3_N_4_) (a bioactive ceramic with antimicrobial properties) embedded particulate coatings affect flexural or fracture behavior? To answer these research questions, we fabricated CCF-PEKK plates with three fiber layups (0°/90°, +45°/−45°, and 0°/90°/+45°/−45°) with the goal of approaching the flexural modulus of cortical bone (1.7–16.3 GPa). Next, half of the hybrid-specimens were spray-coated with submicron Si_3_N_4_ powder. Four-point bending tests demonstrated that fiber orientation significantly influenced flexural modulus and strength. The 0°/90° layup exhibited the highest flexural modulus (67.6 GPa) and strength (1020 MPa), while the +45°/−45° configuration showed the lowest values (15.6 GPa, 217 MPa), but displayed superior load dissipation in axial fiber orientations and was able to reproduce moduli values akin to those of cortical bone range. SEM analysis confirmed uniform Si_3_N_4_ coating distribution, with no observable impact on crack initiation or propagation. No difference (p > 0.01) in flexural modulus or strength was observed between the uncoated and coated specimens, suggesting that Si_3_N_4_ is not associated with static flexural properties of CCF-PEKK. These findings support the feasibility of hybrid-manufactured CCF-PEKK trauma plates as potential alternatives to conventional metallic implants. Further investigations into the long-term fatigue behavior and bioactivity of Si_3_N_4_ coatings are warranted.

## 1. Introduction

While trauma plate fixation has been used for over a century to repair and realign bone fractures, ~10 % of patients suffer from abnormal or delayed healing, frequently leading to implant removal (Einhorn and Gerstenfeld, 2015; Reith et al., 2015; Bostman and Pihlajamaki, 1996; Goharian et al., 2017; Fleischhacker et al., 2024). *In vivo*, metallic plates (titanium [Ti-6Al-4V] and stainless steel) provide initial fracture-site stability. However, their stiff fixation can prevent secondary healing mechanisms from taking place: high strain from the metal implant can restrict the local micromotion necessary to minimize gap separation between the broken bone, forming callus tissue (Kaur and Singh, 2019; Harvin et al., 2017; Ghiasi et al., 2017; Basgul et al., 2020; Ganesh et al., 2005; Kurtz et al., 2022, 2023, 2025). Furthermore, the elastic modulus of metals exceeds that of cortical bone (1.7–16.3 GPa) by over an order of magnitude, contributing to delayed bone healing and in a subset of cases, nonunion (Kaur and Singh, 2019; Niinomi and Nakai, 2011; Uhthoff et al., 2006; Al- et al., 2017; Chmielewska and Dean, 2024; Niinomi, 1998; Panayotov et al., 2016; Kurtz and Devine, 2007) (Motherway et al., 2009; Lee et al., 2019; Auperrin et al., 2014; Smith et al., 2023). Continuous fiber-reinforced polyetheretherketone (CCF-PEEK), with its modulus lower than metallic alloys, was introduced to reduce nonunion. However, for both metal and CCF-PEEK biomaterials, infection remains a major challenge following trauma plate implantation (Darouiche, 2004; Moriarty et al., 2010; Zhang et al., 2023).

Recently, carbon fiber reinforced polyaryletherketone (CFR-PAEK) materials, including CFR-polyetheretherketone (CFR-PEEK) and CFR-polyetherketoneketone (CFR-PEKK) have been translated to the field of additive manufacturing (AM), where technologies such as fused filament fabrication (FFF) can further tailor macro-molecular performance by altering both fiber orientation within the print layer (plie) and stacking sequence (the order in which layers are deposited). For example, Parmiggiani et al., was able to generate CFR-PEEK composites with ranges of 3–24 GPa (flexural stiffness) and 92–341 MPa (flexural strength) when altering layups from a 45°/−45° stacking sequence to a 0° configuration (Parmiggiani et al., 2021). Similarly, when evaluating different layups (0°, +45, 90, 0°/90°, 45°/−45°, 0°/90°/+45°/−45° and 0°/+45°/+90°/–45°) in tension, stiffness ranges 2–24 GPa and strength 46–570 MPa ranges were achieved (Parmiggiani et al., 2021). Lupone et al., recorded tensile modulus values 23.1–48.3 GPa and tensile strength values of 212–598 MPa when assessing the performance of continuous CF/polyamide specimens with different stacking sequences (0°, 0/90°, 0°/+60° and 0°/+45°/90°/−45°) (Lupone et al., 2022). Li et al., built short CF-PEEK specimens in a helicoidal layup configuration and compared their flexural performance to quasi-isotopic variants under different heat treatment (control, 250 °C for 2 and 6 h) methods. The helicoidal specimens recorded flexural stiffness values 23.9 % (~3 GPa – no heat treatment [control]), 13.1 % (3.3 GPa–250 °C at 2 h) and 20.4 % (3.5 GPa–250 °C at 6 h) higher than their quasi-isotopic counterparts. Likewise flexural strength increased 6.9 % (~70 MPa – no heat treatment [control]), 9.3 % (85 MPa–250 °C at 2 h) and 2.8 % (88 GPa–250 °C at 6 h) vs. the quasi-isotopic specimens (Li et al., 2023).

Despite efforts to thermally (heated build chamber, heated build platform and high-temperature hot-end) stabilize PEEK during printing, its high melting temperature (*T*_m_), viscosity and fast crystallization kinetics can sacrifice resolution, geometrical accuracy and mechanical performance (Petersmann et al., 2023; Yi et al., 2021; Basgul et al., 2021; Wang et al., 2019; Pierre et al., 2024; Smith et al., 2024). In contrast, PEKK has a lower *T*_m_ and slower crystallization kinetics, enabling it to be printed at lower temperatures, potentially mitigating the aforementioned print issues (Maloney et al., 2025; Rashed et al., 2022). However, the adoption of load-bearing FFF components is low due to the presence of voids, which reduce mechanical performance and cause unpredictable failure (Jipa et al., 2022; Yadav et al., 2023; Zanjanijam et al., 2020; Arif et al., 2018). Studies performed by Rabinowitz et al., have shown that uniaxial compression molding PEKK-SCF can overcome these issues, where controlling the magnitude and duration that heat and pressure are simultaneously applied uniformly melts the PEKK matrix, filling inclusions and improving adhesion between print layers. Furthermore, controlled cooling encouraged chain packing and densification phenomena, increasing part crystallinity. Consequently, significant rises in flexural moduli were reported (Rabinowitz et al., 2023).

While consolidation, post-FFF printing, isn’t presently suitable for complex-organic-porous three-dimensional geometries (without the availability of expensive molds and tooling to mitigate component damage and deformation), its use is far more easily leveraged to fabricate flat 2.5D denser structures like trauma plates (prior to the insertion of drill-holes). Silicon nitride (Si_3_N_4_), a bioactive ceramic, is an interesting potential coating material for PAEK polymers. It has been reported to encourage osteoblast maturation/mineralization (promoting implant-tissue stability), while displaying anti-microbial behavior in both stock form or when composited into PEEK (Basgul et al., 2024; Du et al., 2023; Bock et al., 2017; Pezzotti et al., 2017). This dual phenomenon is credited to its nitrogen content, creating an amorphous silicon oxynitride layer (~3–5 nm) in aqueous environments, facilitating protein adsorption and osteoblast attachment, whilst releasing ammonia/ammonium by a surface hydrolysis reaction that hinders prokaryotic bacterial metabolism (Pezzotti et al., 2017; Boschetto et al., 2020; Gorth et al., 2012; Katsaros et al., 2022). We hypothesized that the attractive physiochemical properties of Si_3_N_4_ may be translatable as a surface coating for PAEK (Boschetto et al., 2020).

In this study we present the hybrid-manufacture (FFF + uniaxial compression molding) of SCF-PEKK (matrix) embedded with continuous CF tape (reinforcement) to form CCF-PEKK-SCF trauma plates with different plie arrangements. Post-consolidation, we then coated the exterior surface of the plates with Si_3_N_4_ and evaluated their flexural performance. To determine whether Si_3_N_4_ coated CCF-PEKK-SCF are suitable for trauma plate applications we asked the following questions: 1.) Can layup orientation be tuned to replicate the mechanical stiffness of cortical bone in flexion? 2.) How does the structure of the different layups influence fracture behavior? and 3.) Do Si_3_N_4_ coatings affect either flexural properties or fracture behavior?

## 2. Methods

### 2.1. Material fabrication

**Fig. 1.**
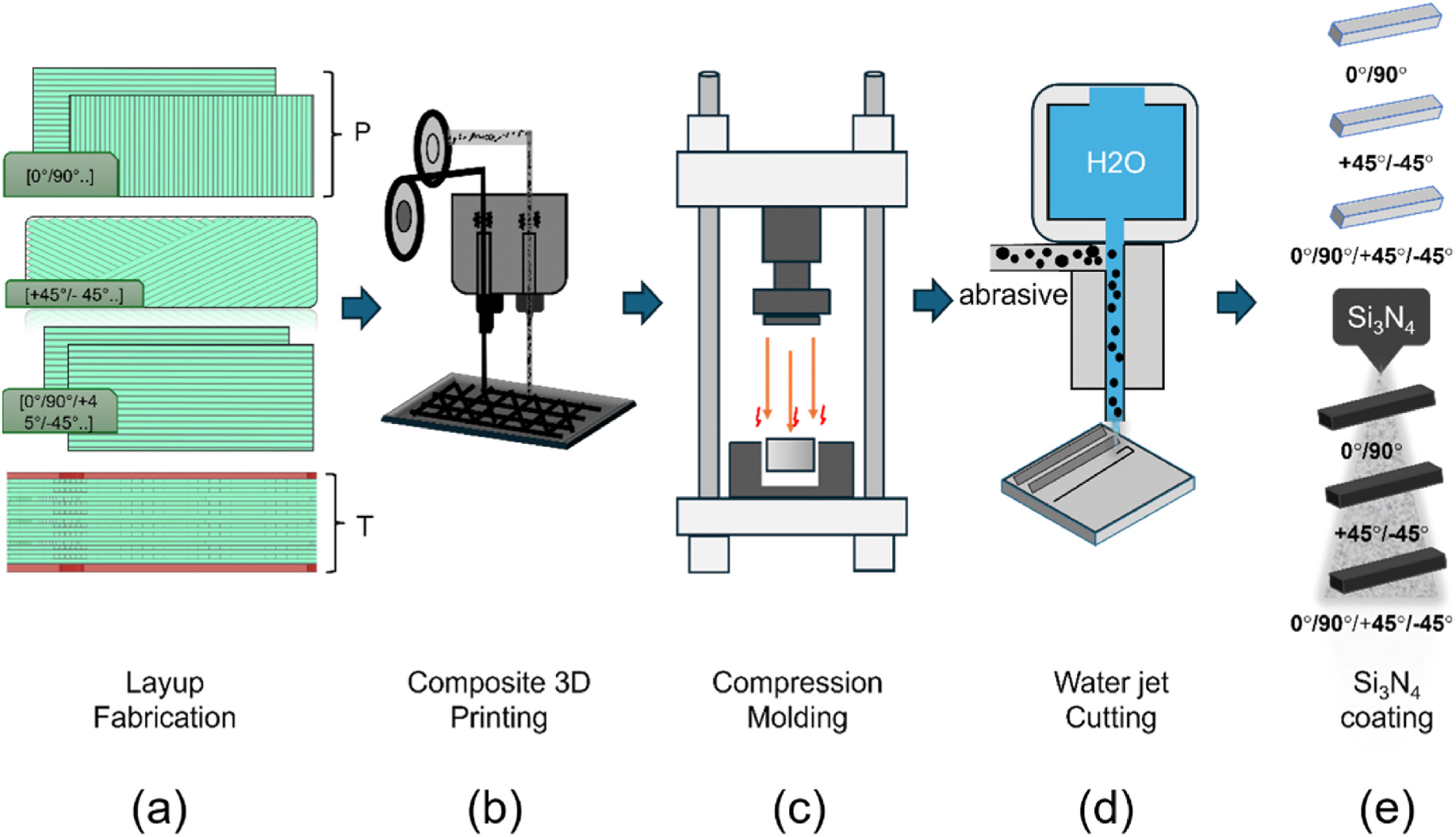
Schematic representation of (a) layups [0°/90°], [+45°/−45°], and [0°/90°/+45°/−45°] in planar view (b) composite 3D printing technology, (c), uniaxial compression molding, (d) water jet cutting and (e) Si_3_N_4_ coating of the CCF-PEKK-SCF specimens.

CCF-PEKK-SCF plates measuring 300 × 150 mm were manufactured by 9T Labs (Zurich, Switzerland) using FFF (Red Series build module 9T Labs, Zurich, Switzerland), followed by uniaxial compression molding (Red Series Fusion module, 9T Labs, Zurich, Switzerland). PAN-based continuous carbon fiber was adopted as the reinforcement element, while short carbon fiber PEKK (weight 8.1 %) was used as the matrix material. The printing and compression parameters of the specimens were not disclosed by 9T labs. The CCF-PEKK-SCF Plates were produced with three alternating layups of continuous carbon fiber 0°/90°, +45°/−45°, and 0°/90°/+45°/−45° (Fig. 1). These plates were then waterjet cut into an average of 13 × 2.77 × 128 mm (about 5.04 in) specimens. For each layup configuration, 48 Specimens were prepared, of these, 24 were spray-coated with Si_3_N_4_ (SINTX Technologies, U.S), and subsequently warm-pressed using a heated uniaxial die to embed the particles into the surface, while the remainder were left uncoated.

### 2.2. Mechanical testing

Four-point bending tests were used to determine the flexural strength and moduli of the CCF-PEKK-SCF specimens in accordance with ASTM D 7264/D 7264M. Specimens were loaded onto four-point bend testing fixtures (Wyoming test fixtures inc. U.S) attached to an electromechanical testing system (Exceed E45, MTS, U.S) equipped with a 30 kN load cell. A support span-to-depth ratio of 32:1 was chosen, where the loading span was half of the support span, with an average sample depth of 2.77 mm. The support span was set to 90 mm, resulting in loading span of approximately 45 mm. These dimensions were selected to maintain the desired span to depth ratio while accommodating the practical constraints of the testing setup. Deflection at the center was measured using a Deflectometer (3540–050M-ST, epsilon technologies, U.S) with a measuring range of 50 mm;

The rate of crosshead motion, R (mm/min), was calculated using the following formula:

Equation (1). Rate of crosshead motion

$$R=\frac{0.167ZL^{2}}{d}$$

Where.

- R is the rate of crosshead motion, mm (in.)/min,
- Z is the straining rate of the outer fiber, mm/mm (in./in.) min. specified as 0.01,
- L is the support span mm (in.), and
- d is the depth of the beam mm (in.)

### 2.3. Statistical analysis

All statistical analysis was performed using GraphPad Prism (version 8.4.3, Boston, MA, USA), Normality for each group (n = 24) was assessed using the Anderson-Darling and Shapiro-Wilk test. As most groups did not meet the assumption for normality (α = 0.05), group comparison for both flexural modulus and peak flexural stress were performed using non-parametric Kruskal-Wallis test, followed by Dunn’s multiple comparisons for post hoc analysis. Statistics were set at p < 0.05.

### 2.4. Fractography

One Si_3_N_4_-coated and one uncoated sample from each layup configuration was subjected to cryogenic fracture under a fume hood, where liquid nitrogen was used as a freezing agent. Once frozen, the samples were manually snapped to create a fracture surface without cutting artifacts. Samples were sputter-coated using an 80/20 Pt/Pd source. The post-fracture cross-section surfaces were then imaged by scanning electron microscope (Supra 50VP field-emission, Zeiss, Germany). Magnifications of 1000× and 5000x were used to examine the surface morphology, with an average working distance of 19.8 mm for uncoated and 18.8 mm for the Si_3_N_4_-coated samples. For post-fracture cross-sectional analysis, a magnification of 300× was employed, using a working distance of approximately 5.5 mm for uncoated and 9.1 mm for coated samples, which enabled a detailed evaluation of fracture mechanics and crack initiation behavior.

## 3. Results

### 3.1. Can layup orientation be tuned to replicate the mechanical stiffness of cortical bone in flexion?

**Fig. 2.**
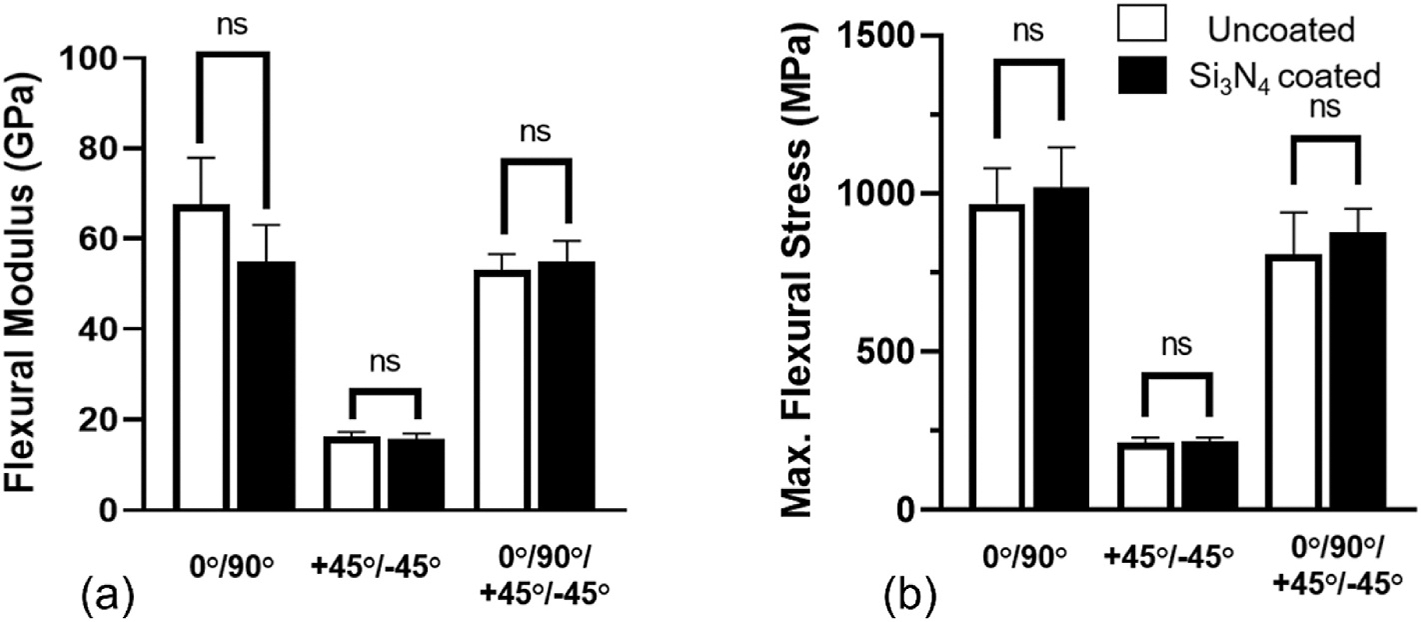
(a) Flexural moduli and (b) flexural strength of uncoated (white) and Si_3_N_4_ coated (black) specimens with 0°/90°, +45°/−45°, and 0°/90°/+45°/−45° fiber lay. ANOVA Krushkal-Wallis Multiple Comparison Test (**P < 0.01; *P < 0.1; ns not significant).

Altering CF layup orientation significantly affected the flexural performance of the CCF-PEKK-SCF plates. 0/90° layup specimens recorded a flexural modulus of 67.6 ± 10.3 GPa, which decreased by 21.3 % (53.2 ± 3.4 GPa) when fibers were re-configured to a 0°/90°/+45°/−45° orientation (Fig. 2 a and A1-appendix). The +45°/−45° specimens recorded the lowest levels of stiffness (16.2 ± 1.1 GPa), which equated to a 76 % and 69.5 % reductions in performance when compared to the 0/90° and 0°/90°/+45°/−45° uncoated specimens, respectively. Recorded maximum flexural stresses were highest in the 0°/90° (967 ± 113 MPa) configuration, followed by 0°/90°/+45°/−45° (810 ± 131 MPa) and lastly +45°/−45° (213 ± 16.3 MPa) in the uncoated specimens (Fig. 2 b and A1-appendix). Of the different layup orientations, only +45°/−45° was able to replicate a stiffness range equivalent to cortical bone. Whereas 0/90° and 0°/90°/+45°/−45° layups exceeded the stiffness of cortical bone by ~3–4x.

### 3.2. How does the structure of the different layups influence fracture behavior of CCF-PEKK-SCF plates?

**Fig. 3.**
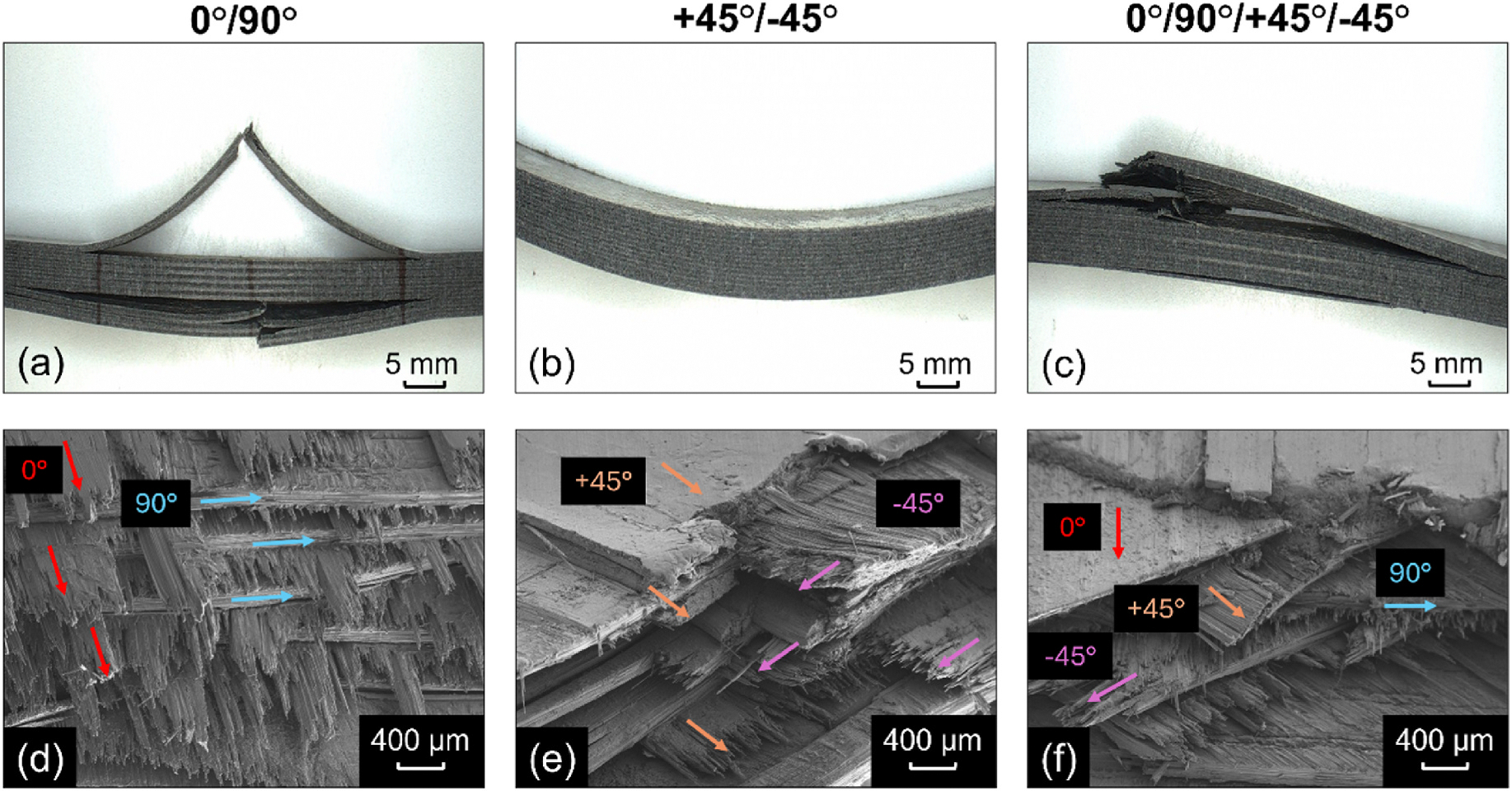
Macroscopic flexural failure modes of uncoated (a) 0°/90°, (b) +45°/−45° and (c) 0°/90°/+45°/−45° CCF-PEKK-SCF specimens post-4-point bend. Scanning electron micrographs of (d) 0°/90°, (e) +45°/−45° and (f) 0°/90°/+45°/−45° fiber layups in uncoated specimens post-freeze fracture.

Photographs representing the macroscopic failure mechanisms of the uncoated 0°/90°, +45°/−45°, and 0°/90°/+45°/−45° CCF-PEKK-SCF specimens are presented in Fig. 3a–c. The 0°/90° specimens displayed interlayer delamination and subsequent brittle fiber fracture in the longitudinal plane, perpendicular to the applied load. Specimens with the +45°/−45° layup configuration did not display any interlaminar delamination between adjacent print layers, however, a more plastic-ductile behavior was seen. The 0°/90°/+45°/−45° had a mixed failure mode, where interlayer delamination and brittle fiber fracture occurred in the upper and lower bulk of the specimen, as seen in the 0°/90° samples. However, breakages in the +45°/−45° layers were also observed.

**Fig. 4.**
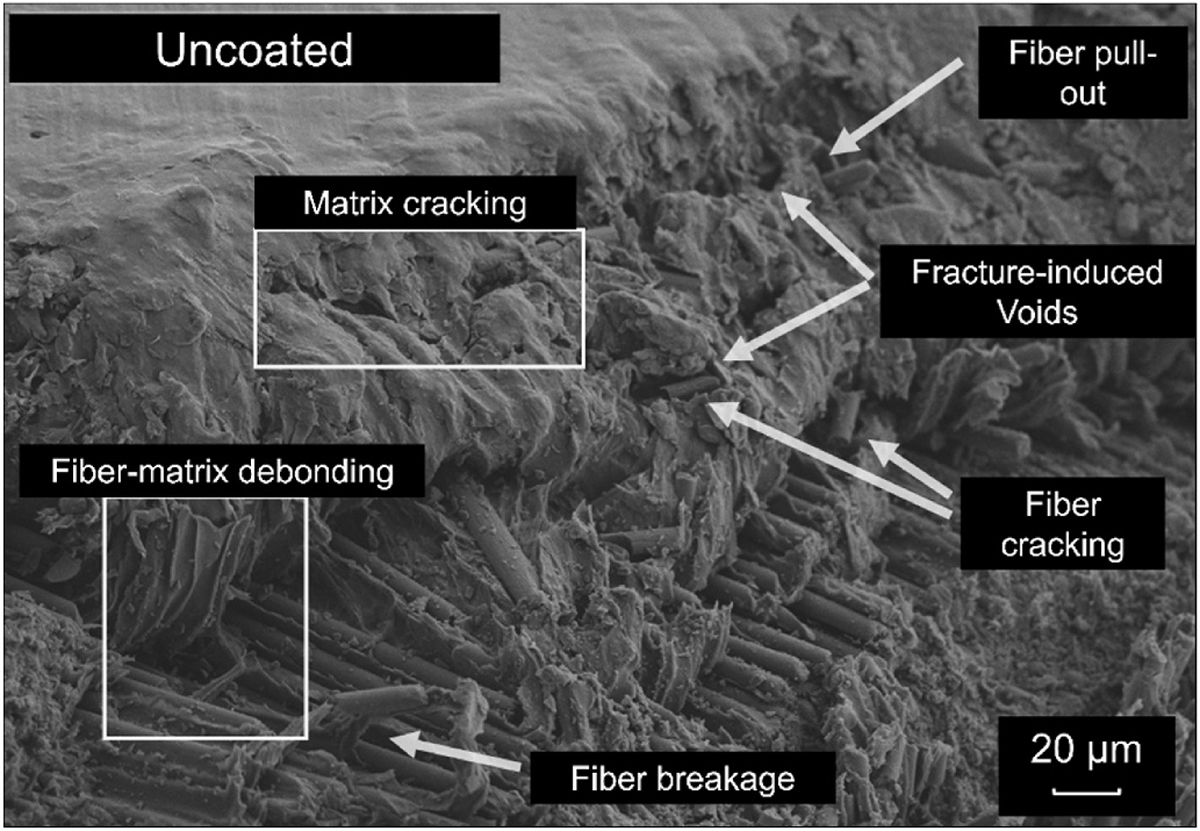
Representative scanning electron micrograph of uncoated +45°/−45° specimen displaying catastrophic failure mechanisms due to freeze-fracture.

SEM was used to assess both the surface morphology and post-fracture cross-sections of the CCF-PEKK-SCF specimens and their layups (Fig. A2-appendix and 3 d–f). The surfaces of all three fiber layups exhibited smooth profiles, displaying striations and negligible irregularities (cavities/bumps) in the microscale range. Post-fracture, each distinct specimen layup was accurately represented in their respective cross-section i.e. 0/90° displayed fibers running in the longitudinal plane (0°), followed by fibers deposited in the transverse plane (90°) in the next sequential print layer (Fig. 3d). The was again true for the +45°/− 45° specimens which showcased fibers deposited at alternating right angles (+45°/− 45°) per print layer (Fig. 3e) and the 0°/90°/+45°/− 45° and +45°/− 45° that displayed repeating stacks of longitudinal, transverse and alternating right angle layups (Fig. 3f). All three layups were repeated throughout the entirety of the specimen builds. All specimen cross-sections exhibited fiber-matrix debonding and matrix cracking, exposing the smooth surfaces of the fibers (Fig. 4). Fiber cracking, breaking and/or pull out were also observed, which left voids across the matrix.

### 3.3. Do Si3N4 coatings affect the flexural strength or fracture behavior of CCF-PEKK-SCF plates?

When evaluating the effect of Si_3_N_4_ coatings on the flexural moduli of the specimens, negligible changes in performance were recorded for the +45°/− 45° (16.2 ± 1.1 GPa) and 0°/90°/+45°/−45° (54.9 ± 4.6 GPa) layups (Fig. 2 a). The 0°/90° Si_3_N_4_ coated specimens (54.9 ± 8.1 GPa) recorded an 18.8 % decrease in stiffness compared to their uncoated (67.6 ± 10.3 GPa) counterparts, however like the other layups, statistically significant (p > 0.01) differences were not observed between each uncoated and coated variant. The maximum flexural stress values recorded were highest in the 0°/90° (1019 ± 127 MPa) configuration, followed by the 0°/90°/+45°/−45° (879 ± 73 MPa) and lastly the +45°/− 45° (217 ± 11.8 MPa) layups (Fig. 2 b). Again, coating the CCF-PEKK-SCF specimens with Si_3_N_4_ did not significantly (p > 0.01) affect maximum flexural stress when compared to their uncoated counterparts.

**Fig. 5.**
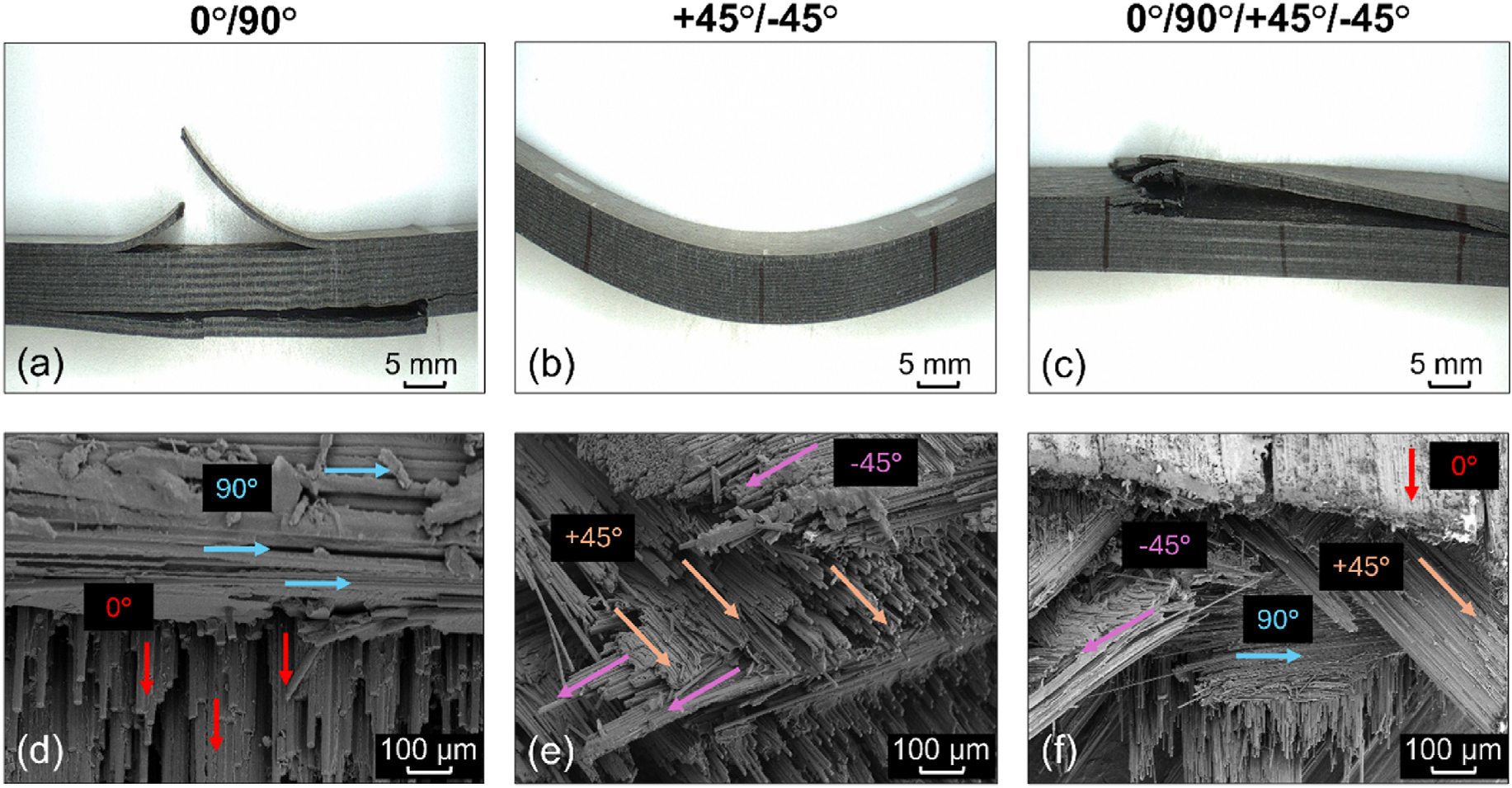
Macroscopic flexural failure modes of Si_3_N_4_ coated (a) 0°/90°, (b) +45°/−45° and (c) 0°/90°/+45°/−45° CCF-PEKK-SCF specimens post-4-point bend. Scanning electron micrographs of Scanning electron micrographs of (d) 0°/90°, (e) +45°/−45° and (f) 0°/90°/+45°/−45° fiber layups in Si_3_N_4_ coated specimens post-freeze fracture.

Macroscopic photographs of the Si_3_N_4_ coated specimens post 4-point bend are presented in Fig. 5a–c. All fiber layups displayed comparable levels of deformation and failure mechanism with respect to their uncoated variants discussed in 3.1.

**Fig. 6.**
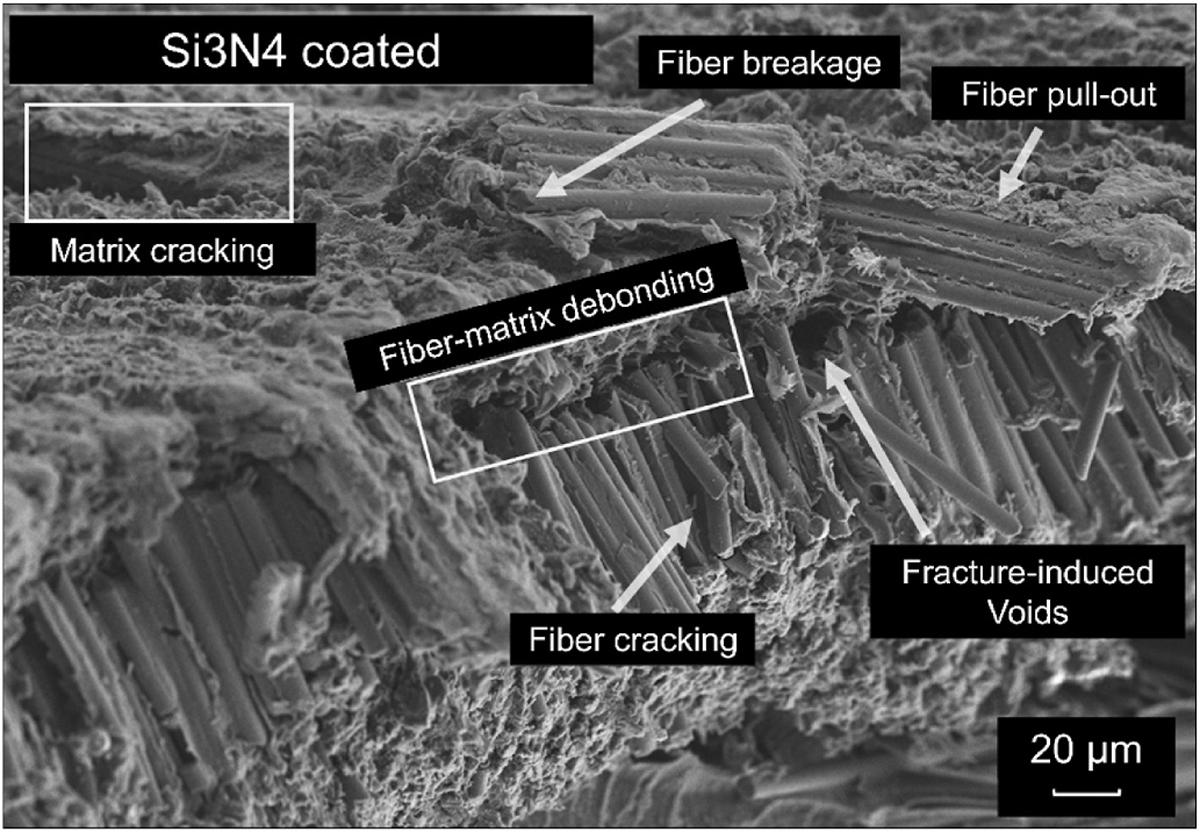
Representative scanning electron micrograph of uncoated +45°/−45° specimen displaying catastrophic failure mechanisms due to freeze-fracture.

The surfaces of the Si_3_N_4_ coated CCF-PEKK-SCF specimens displayed rougher-granular morphologies with an evenly distributed layer of silicon nitride in the nanometer scale (Fig. A2-appendix). The coating was well-adhered, without irregularities, confirming strong interfacing bonding between Si_3_N_4_ and all three CCF-PEKK-SCF substrates. As per uncoated specimens, all fiber layups were accurately represented and repeated throughout the Si_3_N_4_ coated variants (Fig. 5d–f). Likewise, the fracture profiles of the specimens post-freeze fracture displayed fiber-matrix debonding, matrix cracking, fiber exposure, fiber cracking, fiber breaking, fiber pull-out and void formation in the matrix (Fig. 6).

## 4. Discussion

In this study we investigated the effect of CF orientation (0°/90°, +45°/−45° and 0°/90°/+45°/−45°) and Si_3_N_4_ surface coatings on the flexural performance and fracture behavior of hybrid manufactured (FFF + uniaxial consolidation) CCF-PEKK-SCF plates to determine their suitability for trauma plate applications. Continuous CF orientation had a significant effect on both the modulus and peak stress of the plates, with the 0°/90° layup recording moduli values ~4.2x and ~1.3x that of the 45°/−45° and 0°/90°/+45°/−45° respectively. Stiffness disparity was credited to the manner in which the different layups dissipated load with respect to loading direction. Half of the 0°/90° specimen’s structure contained 0° fiber layups (orientated perpendicular to loading) which are highly efficient at resisting axial and bending loads (Li et al., 2019). This enabled comparatively higher stresses to be dissipated along the continuous fiber lengths, restricting matrix deformation and micro-crack propagation (specifically in the 0° layers) at a greater rate than the 45°/−45° and 0°/90°/+45°/−45° layups (Wang et al., 2020; Mohamed and Abdelbary, 2023). Despite the 0°/90° layup withstanding the highest level of load transmission, their flexural moduli exceeds cortical bone range. Which in turn, could promote stress shielding behavior over time.

The 45°/−45° layups reported the lowest moduli, which was credited to reductions in fiber length, reductions in fiber-matrix interface length and the addition of localized off-axis rotational forces acting on the structure itself (Mohamed and Abdelbary, 2023). In turn, this caused stresses to build up between the matrix and the fiber far faster than those deposited longitudinally (0°/90°), despite the balanced layup of the structure (Mohamed and Abdelbary, 2023). Consequently, plate stiffness and strength was reduced, whilst ductility increased with respect to the other layups. Parmiggiani et al., reported similar findings where 45°/−45° CFR-PEEK specimens recorded the lowest levels of stiffness (3.3 GPa) and strength (92 MPa) vs. 0° (24.4 GPa and 341 MPa), 0°/90° (14.6 GPa and 241 MPa) and 0°/90°/+45°/−45° (10.3 GPa and 224 MPa) and had to be bent by 180° press to induce fiber fracture post-4-point bend (Parmiggiani et al., 2021). While flexural stiffness was the lowest of the three layups, the 45°/−45° specimens were the only ones to match cortical bone moduli range. Furthermore, its plastic-ductile deformation pathway is considered advantageous for trauma plate applications (vs. brittle failure seen in 0°/90° and 0°/90°/+45°/−45°), where if mechanical failure were to occur, continuous CF would not be exposed to the body. In turn, potentially preventing any adverse local tissue reaction and/or resection surgery.

The 0°/90°/+45°/−45° layup recorded intermediate levels of performance, despite being isotopically stacked i.e. theoretically the combination of a 0°/90°/+45°/−45° layup has the capacity to dissipate loading equivalent to the 0°/90° layup (Gibson, 2016). However, upon the onset of plastic deformation maximum stress significantly declined. This was attributed to reductions in the number of 0° fiber layups within the structure which have the greatest capacity to dissipate tensile-compressive and shear forces acting on the structure, as acknowledged in prior findings (Parmiggiani et al., 2021; Corum, 2002), coupled with the addition of the +45°/−45° layups (within the structure) that contributed to rotational stress accumulation at the fiber-matrix interface, reducing post-yield performance (Wisnom, 2012). Like the 0°/90° layups, the 0°/90°/+45°/−45° specimens displayed moduli values that exceed cortical bone as well as failure in a catastrophically brittle manner. Hence, making them less suitable for trauma plate applications.

In addition to the dominant performance roles of the continuous CF, the short carbon fibers (distributed throughout the PEKK matrix in all layup strategies) also enhanced the overall mechanical performance (stiffness, strength and fracture resistance) of the composite structures by improving load transfer and inhibiting crack propagation (Yang et al., 2021; Guan et al., 2020; Zhang et al., 2022; Ramaswamy et al., 2023). Striking a balance of short carbon fiber to PEKK ratio is critical in ensuring that maximum levels of wettability and mechanical performance are achieved between the two materials (and the continuous CF) whilst preventing over saturation of the matrix that can lead to embrittlement and early onset component failure (Xi et al., 2018; Peng et al., 2021).

Layup orientation had a significant influence on the fracture behavior of the specimens, where those containing the 0° fibers displayed delamination between print layers (as consequence of high compressive and tensile forces), prior to catastrophic fracture of the fibers themselves. Delamination and fiber fracture did not occur in the +45°/−45° layups, instead, the specimens experienced plastic-ductile warpage as result of localized off-axis rotational forces on their fibers.

In the freeze fractured 0°/90° specimens, fracture occurred through fiber pull-out and matrix cracking, indicating strong fiber-matrix adhesion and effective load transfer prior to catastrophic failure (Dai et al., 2022; Lin et al., 2023). The presence of fiber pull-out also suggests that despite strong adhesion, localized stress concentrations at the fiber-matrix interface led to debonding and void formation (Rabinowitz et al., 2023; Komal et al., 2025; Sayam et al., 2022). This fracture profile aligns with findings by Rabinowitz et al., who highlighted similar phenomena, where uniaxial compression molded PEKK-SCF plates displayed greater brittle-like failure vs. non pressed specimens which showed brittle-ductile performance. Furthermore, cross-sectional fracture revealed jagged fibers surfaces and strong interlayer bonding between adjacent print layers which was true of this study (Rabinowitz et al., 2023).

The freeze-frozen +45°/−45° specimens displayed fiber-matrix debonding as consequence of reduced fiber length and matrix polymer (deposited in the transverse direction), succumbing earlier to interfacial shear-torque loading. As result, this led to crack propagation, fiber pull-out and fracture induced voids which are indicative of lower shear load transfer between piles. This failure mechanism aligns with previous literature, that shear-dominated laminates exhibit increased susceptibility to delamination due to weaker interfacial bonding (Wisnom, 2012; Morioka and Tomita, 2000).

The isotropic 0°/90°/+45°/−45° layup displayed a combination of the failure mechanisms observed in the unidirectional (0°/90°) and shear dominated (+45°/−45°) configurations. SEM analysis revealed the fiber breakage, fiber pull-out, and matrix cracking (but to a lower degree than the 0°/90° layup), indicating improved load distribution. Additionally, fewer fracture induced voids were present compared to +45°/−45° specimens, suggesting that the alternating fiber orientations provided better resistance to delamination. The presence of fiber-matrix debonding in these specimens were similar to those observed in the other layups, though crack propagation pathways appeared more complex, which is characteristic of a quasi-isotropic laminate (Kumar et al., 2024). This observation is consistent with prior studies on hybrid-laminate systems, where a balanced fiber architecture improves damage tolerance while maintaining sufficient mechanical integrity under flexural loads (Hasanuddin et al., 2023; Liang et al., 2024).

The Si_3_N_4_ surface coating did not significantly impact the flexural modulus or flexural strength of the CF-PEKK layups. Rationale was credited to the fact that the ceramic particles were coated onto the CCF-PEKK-SCF post-consolidation and post-solidification. Consequently, this mitigated the ceramic from entering both the bulk phases of the composite, preventing particle coalescence that can lead to microcavity formation and poor interlaminar wetting between themselves and the molten PEKK matrix (Smith et al., 2021). Such phenomena was seen by Smith et al., who witnessed enhanced levels of particle agglomeration, improper wetting and cavitation when increasing ratios of PEEK particle filler were introduced into a polydimethylsiloxane matrix, when exploring novel 3D-printable composites for biomedical applications (Smith et al., 2021).

The findings from this study support the idea that the hybrid-manufacturing method of FFF and uniaxial compression molding can successfully produce CCF-PEKK-SCF plates with tunable levels of flexural modulus and strength. Of the fiber layups investigated, both uncoated and Si_3_N_4_ coated +45°/−45° layups displayed stiffness values (uncoated = 16.2 ± 1.1 GPa and Si_3_N_4_ coated = 15.6 ± 1.3 GPa) within the accepted range of cortical bone (1.7–16.3 GPa) in flexure, while strength values were exceeded (Motherway et al., 2009; Lee et al., 2019; Auperrin et al., 2014; Smith et al., 2023). The reason for this finding was credited to the quasi-orthotropic nature of the +45°/−45° layup, which better mimicked the anisotropy of cortical bone, providing a balance of plate stiffness and flexibility in pure bending. Further fiber layup fine-tuning could be undertaken to develop a range of CCF-PEKK-SCF plates that can specifically match the performance of bone at different anatomical locations, potentially improving healing rate, while mitigating stress-shielding. Si_3_N_4_ coatings had no significant effect on CCF-PEKK-SCF plate flexural performance, whilst potentially offering additional antimicrobial and bioactive properties. Consequently, this could help promote faster fracture site healing (than without the coating), while reducing the risk of infection and resection surgery. Prior to determining such ideals, the cyclic performance of the coated plates should be undertaken to replicate loading methods expected to be experienced in-situ, as well as biocompatibility studies to determine the effect of the Si_3_N_4_ coatings on the osteoconductivity, osteoblast proliferation and antimicrobial ability of CCF-PEKK-SCF plates.

## 5. Conclusion

Hybrid-manufacturing (3D-printing [FFF] + uniaxial compression molding) methods were used to fabricate CCF-PEKK-SCF composites with different laminate plie orders, prior to their exteriors being coated with Si_3_N_4_. These samples were then tested in 4-point bending. Those printed with a 0°/90° layup exhibited the highest flexural modulus and strength due to efficient load transfer along fiber axes, but their stiffness and strength significantly exceeded that of the cortical bone they were aiming to replace. 0°/90°/+45°/−45° layups displayed isotropic levels of performance but experienced a decline in flexural strength upon plastic deformation when compared to the 0°/90° layup. Again, stiffness and strength values were beyond those of cortical bone, whereas +45°/−45° layups showed reduced stiffness as consequence of early onset interlaminar shear, attaining flexural moduli values representative of acceptable cortical bone range. Si_3_N_4_ coatings had no significant effect on the mechanical performance of the composites which was further confirmed by SEM, where all specimens experienced fiber-matrix debonding, fiber pull-out, fiber breakage, and fracture-induced voids. The findings from this study suggest that hybrid-manufactured CCF-PEKK-SCF coated with silicon nitride warrants further study as a candidate biomaterial for trauma plate applications.

## Supplementary Material

Appendix A. Supplementary Data.docx

Appendix A. Supplementary data

Supplementary data to this article can be found online at https://doi.org/10.1016/j.jmbbm.2025.107141.

## Data Availability

Data will be made available on request.
