# Supplementary material for: Hybrid-manufactured silicon nitride coated CFR-PEKK: A candidate biomaterial for trauma plate applications?
Source: J Mech Behav Biomed Mater. Author manuscript; Available in PMC 2025 Aug 16. (PMC12357486; doi:10.1016/j.jmbbm.2025.107141)
Supplement: Appendix A. Supplementary Data.docx [file NIHMS2100491-supplement-Appendix_A__Supplementary_Data_docx.docx]

**Appendix A. Supplementary data**


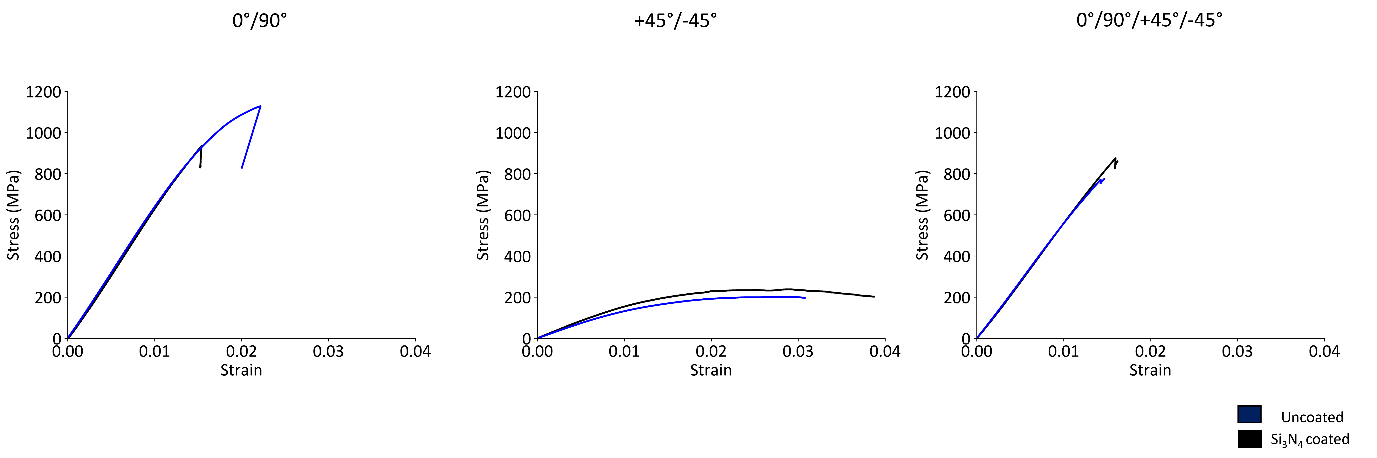

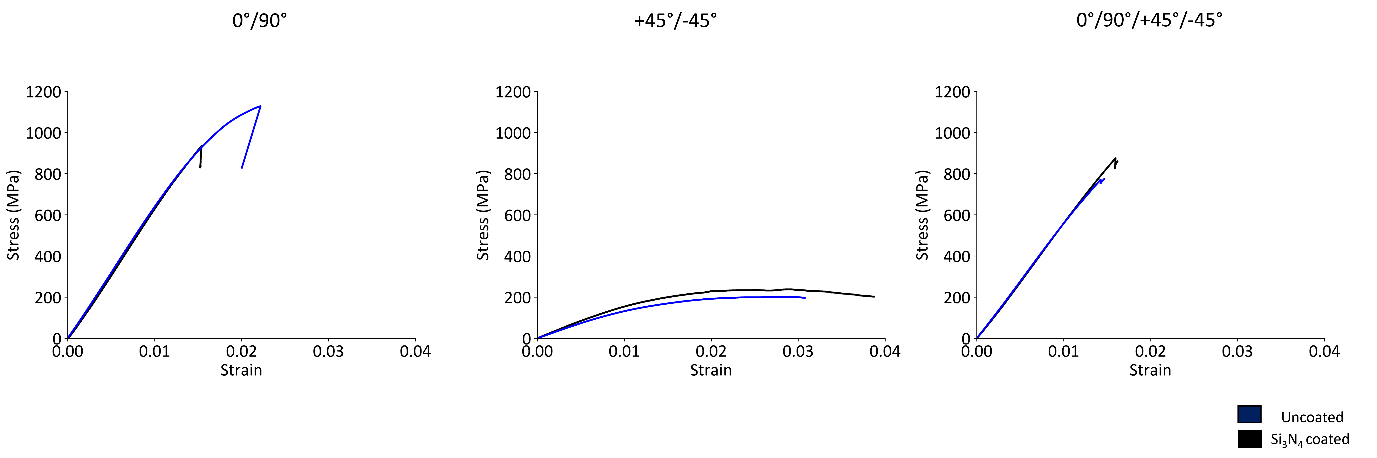

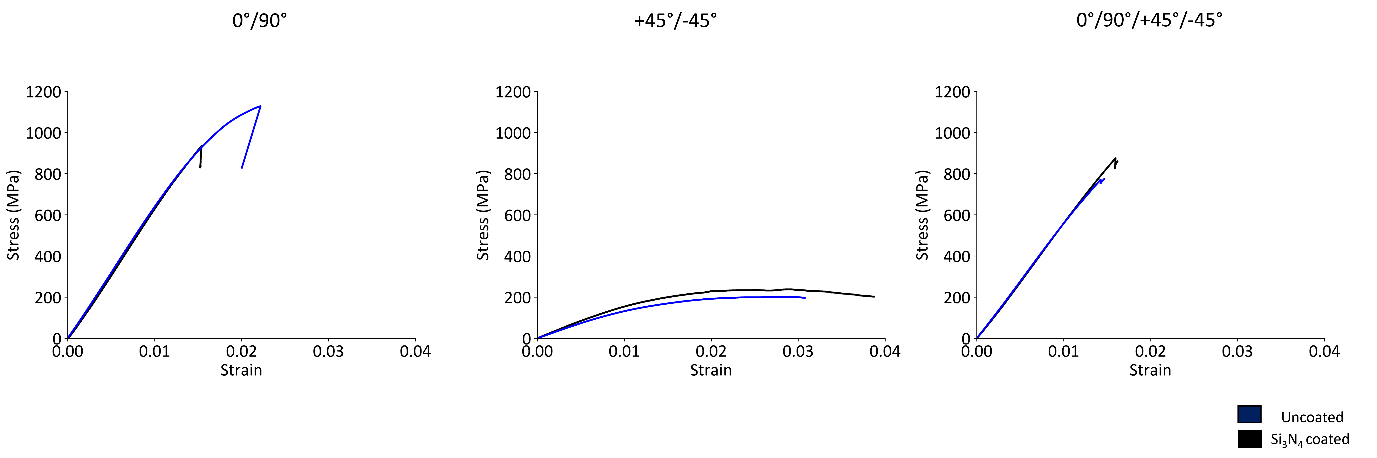


(a)

(b)

(c)

**Figure A1.** Representative flexural stress-strain curves for uncoated and Si₃N₄-coated samples across all layup configurations (a) 0°/90°, (b) +45°/-45 and (c) 0°/90°/+45°/-45 under four-point flexural loading.


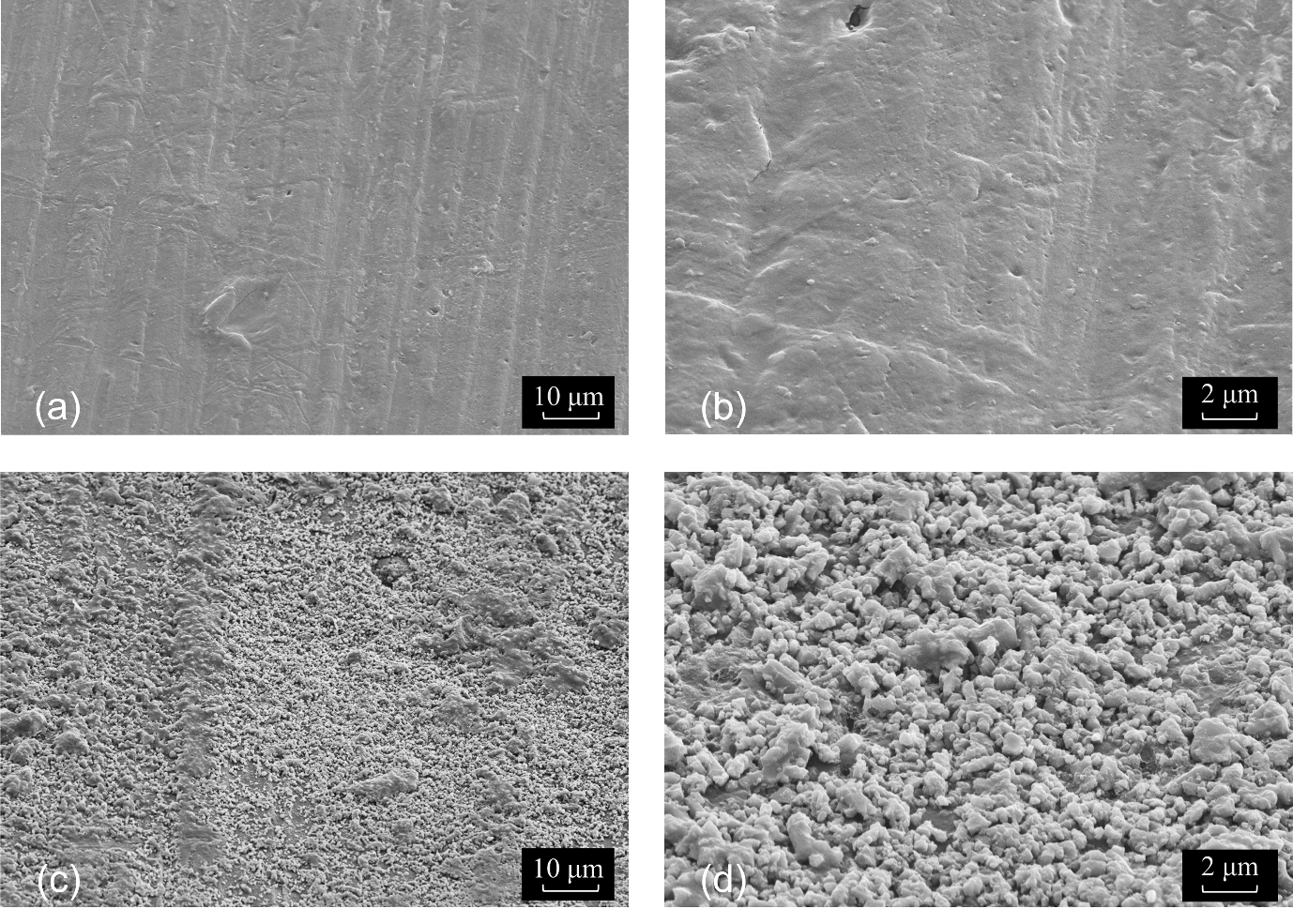


**Figure A2.** Scanning electron micrographs displaying the surface morphology of (a & b) uncoated and (c & d) Si_3_N_4_ Coated laminates.
